# Detailed Soluble Proteome Analyses of a Dairy-Isolated *Enterococcus faecalis*: A Possible Approach to Assess Food Safety and Potential Probiotic Value

**DOI:** 10.3389/fnut.2019.00071

**Published:** 2019-05-17

**Authors:** Simona Cirrincione, Bernd Neumann, Daniela Zühlke, Katharina Riedel, Enrica Pessione

**Affiliations:** ^1^Department of Life Sciences and Systems Biology, Univerity of Torino, Turin, Italy; ^2^Department for Microbial Physiology and Molecular Biology, University of Greifswald, Greifswald, Germany

**Keywords:** foodborne bacteria, gel-free proteomics, virulence, antibiotic resistance, host interaction

## Abstract

Enterococci are common inhabitants of the gastrointestinal tracts of humans and animals and thanks to their capability to tolerate different environmental conditions and their high rates of gene transfer, they are able to colonize various ecological niches, as food matrices. *Enterococcus faecalis* bacteria are defined as controversial microorganisms. From one side they are used as food starters, bio-control agents and probiotics to improve human or animal health. From the other side, in the last two decades enterococci have emerged as important nosocomial pathogens, because bearing high-level of resistance to antibiotics and several putative virulence factors. In this study, the soluble proteome quantitation data (LC-MS/MS) of the food-isolated strain *E. faecalis* D27 (dairy-isolate) was compared with the soluble proteome quantitation data of the pathogenic *E. faecalis* UW3114 (urinary tract infection isolate) and with the one of the health promoting strain *E. faecalis* Symbioflor1, respectively. The comparison of cytosolic protein expression profiles highlighted statistically significant changes in the abundance of proteins mainly involved in specific metabolic pathways, nutrient transport, stress response, and cell wall modulation. Moreover, especially in the dairy isolate and the clinical isolate, several proteins with potential pathogenic implications were found, such as serine proteases, von Willebrand factor, serine hydrolase with beta lactamase activity, efflux transporter, and proteins involved in horizontal gene transfer. The analysis of the extracellular proteome provided interesting results concerning proteins involved in bacterial communication, such as pheromones and conjugative elements and also proteins able to interact with human components. The phenotypic characterization evaluating (i) biofilm formation (ii) hemolytic activity on blood agar plates (iii) protease activity (iv) gelatinase (v) antibiotic resistance pattern, enabled us to elucidate the risks associated with the poor characterized foodborne *E. faecalis* D27.

## Introduction

Enterococci are ubiquitous gram-positive bacteria that can be found in various ecosystems, ranging from soil, surface waters, plants, the gastrointestinal tract (GIT) of animals and humans as well as in foods. Enterococci also emerged as important pathogens, since they are one of the major cause of both nosocomial and outpatient-associated infections (1), including clinical manifestations such as urinary tract infections, endocarditis, primary bacteremia and meningitis (2).

In the last decades, enterococci were widely studied because they have intrinsic (absence of target, impermeability, absence of uptake mechanism) and, often, acquired (antibiotic degrading or modifying enzymes) resistance to antibiotics (3, 4). Generally, intrinsic/natural resistance is chromosomally-encoded and therefore non-transmissible, whereas acquired resistance can be plasmid-mediated and hence transmissible by genetic recombination (5). An increasing number of multidrug resistant enterococci has been detected as the predominant microbiota under antibiotic pressure, predisposing hospitalized patients to severe infections and mortality (6).Some virulence factors and antibiotic resistances determinants are carried on mobile genetic elements (MGE) (7), supporting the theory of enterococci as reservoir for antibiotic resistance determinants found in pathogens (8, 9). A large number of reports has focused on the presence or absence of virulence determinants in enterococcal isolates from different origins (food and living being) (10, 11). In particular, the higher incidence in clinical isolated enterococci of genetic elements encoding for virulence factors indicate that these genes enhance their ability to colonize humans, increasing the infection level, as suggested by virulence studies on bacterial mutants in animal models (12).

Within enterococci species, whereas *E. faecium* harbors more antibiotic resistance traits, *E. faecalis* shows a higher potential for virulence, because it synthesizes many proteins that facilitate interaction with environmental (both biotic and abiotic) surfaces, biofilm formation and host colonization (13, 14). Some of these proteins, such as aggregation substance, cytolysin, enterococcal surface protein, gelatinase and protease, are considered to be potential virulence factors (15, 16).

*Enterococcus faecalis* appears to be typically associated with the human GIT (17) and it is one of the first lactic acid bacteria (LAB) to colonize the intestine of the newborn (18). In healthy human GIT, this bacterium is harmless and present in low abundance, however it can cause life-threatening infections during antibiotic-induced dysbiosis (19). *E. faecalis* is usually carried by food, due to its widespread distribution in the environment. In particular it is often isolated from food of animal origin such as meat (20), milk (21), and cheese (22). Its presence may indicate a natural contamination during manufacturing processes, in some cases as a consequence of fecal contamination caused by poor hygiene (23). Otherwise, *E. faecalis* is used as starter culture (24) to carry out fermentative processes, indeed it plays an important role in cheese ripening and aroma development. However, some authors have reported the presence of antimicrobial resistance and virulence determinants in enterococci found in foods, including cheeses (25, 26). Therefore, a food contaminating *E. faecalis* could represent a possible intermediate vehicle for the transmission of pathogenic traits. In this case, bacteria could act as vectors for the dissemination of antibiotic resistance determinants and virulence factors via the food chain to the consumer, a risk that has so far been poorly addressed (27–29). Actually, it has been observed that human gut enterococci are probably acquired from food since they possess the same antibiotypes and toxinogenic profiles than cattle-, pig-, and sheep- isolated enterococci (30).

Despite the risks associated to *E. faecalis* have led to define these bacteria as controversial (31) they are currently present not only in fermented food but even used as health supplements and probiotics by the pharmaceutical industry. Enterococcal probiotics are usually utilized as “food supplements” in the form of encapsulated or lyophilized pharmaceutical preparations (32). This choice is partly motivated by the lower acid resistance of enterococci compared to lactococci and lactobacilli, that render them less suitable to survive the gastric pH transit. These bacteria are thus ingested in high number to achieve functional or probiotic effects especially for treatment of diseases such as irritable bowel syndrome, diarrhea, antibiotic associated diarrhea, or for health improvement such as cholesterol levels lowering or immune regulation (33, 34).

For all these reasons (possible pathogenicity/virulence determinants or transferable antibiotic resistance characters), a careful selection of *E. faecalis* strains for food supplementation should be done and a constant monitoring on new food-isolated enterococcal strains is required to limit the propagation of virtually harmful microorganism. However, evaluating these risks only by genome analysis of strains is limiting since gene function annotation is based just on sequence homologies with genes present in databanks. Therefore, some evidences should be validated by functional investigations as well as by studying bacterial protein profiles and separately analyze sub-proteomes.

In this view, the present study intended to characterize *Enterococcus faecalis* D27, isolated from cheese, with the specific aim to elucidate if this strain harbors pathogenic traits or rather reveals probiotic features. For this reason, we also examine both a pathogenic (clinically isolated) and a probiotic strain belonging to the same species, to understand the similarities and differences among all the three strains by comparing phenotypic traits, proteomic, and biochemical profiles.

## Materials and Methods

### Bacterial Strains and Cultured Condition

In this study three strains of *Enterococcus faecalis* (present in our collection), with different origin were investigated: D27 (isolated from an artisanal cheese), Symbioflor 1 (commercial probiotic strain) and UW3114 (clinical isolated strain, bearing the pathogenicity island of *E.faecalis* MMH594) (35). All strains were maintained in BHI medium (Oxoid, Munich, Germany) at −20°C in 0.5 mL aliquots with 0.5 mL 40% v/v glycerol. For both proteome analyses and phenotypical assays three biological replicates were performed. Bacterial cultures were grown in the BHI medium at 37°C with slight agitation (180 rpm). Culture supernatants (extracellular proteome) and cells (cytosolic proteome) were recovered by centrifugation (10,000xg, 10 min, 4°C) from the same bacterial culture in the early stationary growth phase.

### Sample Preparation for Proteomic Analyses

#### Cytosolic Proteins

The intracellular protein extract was obtained as previously described by Zühlke et al. (36). Briefly, cell pellet was washed with TE buffer and lysed by glass beads (diameter: 0.1–0.11 mm) using a Precellys 24 Homogenizator (Peq Lab, Erlangen, Germany).In-solution digestion of protein extracts with trypsin was done according to the method described previously (37). Proteins were dissolved in 50 mM TEAB/0.1% RapiGest™ SF (Waters, Milford, MA, USA), reduced with TCEP (tris-(2-carboxyethyl)phosphine hydrochloride, Invitrogen, Carlsbad, CA, USA) for 45 min at 60°C and alkylated with iodoacetamide (Sigma, Steinheim, Germany) for 15 min at room temperature. Proteins were digested with 0.5 mg trypsin in a 1:200 ratio (Promega, Madison, MA, USA) for 6 h under gentle agitation at 37°C. Desalting of peptides was achieved using a standard protocol (38). For absolute quantification, a tryptic digest of yeast alcohol dehydrogenase (ADH1, Waters, USA) was added into the samples to final concentration of 50 fmol/μL.

#### Extracellular Proteins

The extracellular protein extract was obtained as previously describe by Lassek et al. (39). Briefly, 10% TCA were added to the cell free supernatant and incubated o/n at 4°C. Samples were centrifuged (10,000 x g, 1 h, 4°C). Protein pellet was washed with 70% ethanol (in the last step 96% ethanol was used). After drying using a vacuum centrifuge (Concentrator plus, Eppendorf) proteins were resuspended in 8M urea/2M thiourea buffer and centrifuged (16,000 x g, 10 min, RT) to recovery the supernatant. Proteins were separated using a Criterion™ TGX™ precast gel (4–20%, 12+2 Well Comb, 45 μL, 1 mm) (Biorad, Hercules, CA, USA), fitting with Mini Protean II-Apparatus (140 V for 75 min, in glycine running buffer). The digestion of the extracellular proteins was performed as described by Lassek et al. (39). Briefly, the excised gel pieces, from SDS-PAGE, were destained using 50% (v/v) methanol in 100 mM NH_4_HCO_3_. Gel pieces were dehydrated and modified trypsin (sequencing grade, Promega, Fitchburg, WI) was added to a final ratio of 1:10 (trypsin/sample) in 50 mM Tris/HCl, pH 7.5, and the sample incubated at 37°C overnight. Peptides were iteratively extracted from the gel using an ultrasonic bath for 15 min.

The protein concentration of both intracellular and extracellular extracts was determined using Roti Nanoquant (Roth, Germany) according to the manufacturer's instructions.

### MS Analysis

#### Cytosolic Proteins

The separation of the peptide mixture from intracellular proteins was performed using a nanoACQUITY™ UPLC™ system (Waters, USA) following the protocol described by Zühlke et al. (36). The obtained data were searched against a randomized *E. faecalis* OG1RF database (NCBI, version 2015-09-01) with added laboratory contaminants and yeast ADH1 sequence and sequences from the proteins encoded by the pathogenicity island of *E. faecalis* MMH594 (5,490 entries). For positive protein identification the following criteria had to be met: 1 fragment ion matched per peptide, 5 fragment ions matched per protein, 1 peptide matched per protein; 2 missed cleavages allowed, primary digest reagent: trypsin, fixed modification: carbamidomethylation C (+57.0215), variable modifications: deamidation N, Q (+0.9840), oxidation M (+15.9949), pyrrolidonecarboxylacid N-TERM (−27.9949). The protein false discovery rate (FDR) was set to 5%. For the final analyses, only identifications based on at least two peptides were considered. A protein had to be identified in at least two out of three technical replicates per biological replicate. In addition, for the final analysis protein had to be present in two out of three biological replicates per time point, which reduced FDR on protein level to <0.7%.

#### Extracellular Proteins

Peptide mixtures resulting from in-gel tryptic cleavage were separated by RP chromatography using an EASY-nLC (ThermoFisher Scientific, Waltham, USA). Fractionated peptides were loaded onto the analytical column at a flow rate of 700 nL min−1 in 100% buffer A (0.1% acetic acid) and separated using a binary 87-min gradient from 5 to 35% of buffer B (0.1% acetic acid in acetonitrile) at a constant flow rate of 300 nL min−1. The EASY-nLC was coupled to an LTQ Orbitrap mass spectrometer (Thermo Fisher Scientific, Waltham, MA, USA). After a full survey scan in the Orbitrap (m/z range from 300 to 2,000, resolution 30,000, lock mass option-enabled [lock mass 445.120025)] MS/MS experiments in the LTQ XL were performed for the six most abundant precursor ions (CID). Unassigned charge states and singly charged ions were excluded from fragmentation; dynamic exclusion was enabled after 30 s. For protein identification, spectra were searched against a database of *E. faecalis* OG1RF (NCBI, version 2015-09-01) containing sequences of all predicted proteins from its genome, including reverse sequences and sequences of common laboratory contaminants and sequences from the proteins encoded by the pathogenicity island of *E. faecalis* MMH594 (5488 entries). Database searches using Sorcerer SEQUEST (version v. 27 rev. 11, Thermo Scientific) and Scaffold 4.4.1 (Proteome Software, Portland, OR, USA) as well as statistical analysis was done as described earlier (40).

### Quantification Methods for Proteome Analysis of *E. faecalis* Strains

#### Cytosolic Proteins

The protocol that was used for the extraction of cytosolic proteins does not involve any steps that might lead to loss of proteins and is compatible with downstream in-solution digestion of proteins. For this reason, the label-free LC-IMSE approach was chosen for identification and quantification of proteins to reliably determine the abundances of cytosolic proteins. In particular, the absolute quantitative data were obtained by applying a LC-IMSE approach in combination with the Hi3 quantification method (41, 42).

#### Extracellular Proteins

Extraction of extracellular proteins is based on precipitation and subsequent solubilization of proteins in a buffer that is not compatible with in-solution digestion. Therefore, separation by SDS-PAGE and in-gel digestion of proteins was applied, allowing to obtain reliable quantitative results based on relative spectral abundances. Relative quantitative data were obtained using a label-free LC-MS/MS approach and using normalized spectral counts (NSAF) for quantification of identified proteins (43). In this approach, the spectral counts of a protein are divided by its length and normalized to the sum of spectral counts/length in a given analysis.

### Database Deposition and Graphic Representation of Data

The mass spectrometry proteomics data have been sent to the Proteome Xchange Consortium via the PRIDE partner repository (44) with the dataset identifier PXD011701 for the intracellular proteins and identifier PXD011660 for the extracellular proteins. For functional prediction and classification of both cytosolic and extracellular proteins, the analysis pipeline Prophane was used (40, 45) and Voronoi treemaps were generated using Paver (Decodon, Greifswald, Germany; http://www.decodon.com/paver/). Thanks to the Voronoi treemaps, it is possible to visualize the bacterial proteome, grouping the proteins according to their function, sub-function or specific protein name (the functional prediction is based on TIGRFAMS and Cluster of orthologous groups (COG).

### Phenotypic Analyses

The *E. faecalis* isolates were analyzed for activity of different known virulence factors namely, biofilm formation, gelatinase, protease, and hemolytic activity.

#### Protease Assay

The assay was performed on the cell-free supernatant as described by Pessione et al. (16), using Azocasein solution (1% Azocasein, 50 mMTris-HCl, pH 7.5, 5 mM EDTA) instead of Azoalbumin.

#### Gelatinase Assay

The assay was performed on the cell-free supernatant as described by Pessione et al. (16). Briefly, 4 ml aliquots of a solution of 3% (p/v) gelatine in 50 mM pH 7.3 Tris-HCl were distributed in 15 ml tubes. At room temperature gelatin solution appears to be liquid, while at 4°C it becomes solid. The tubes were incubated at 4°C for 1 h to evaluate the ability of solution to solidify and then incubated at 37°C to bring it at the liquid state again. Four hundred microliters of the concentrated bacterial extracellular extracts were added to the liquid solutions and the mixtures were incubated 2 h at 37°C, the optimum for gelatinase activity. After that, they were incubated overnight at 4°C to allow to gelatin to solidify again if gelatinase was not present in the extracellular extracts.

#### Hemolytic Assay

Cells from a liquid culture were plated on Columbia Agar base (Oxoid, Munich, Germany) plates enriched with 5% defibrinated horse blood (TCS Biosciences, Botolph Claydon, UK). The hemolytic activity was checked after 48 h examining the cultures for signs of β-hemolysis (clear zones around colonies), α-hemolysis (green zones around colonies), γ-hemolysis (no sign of hemolysis).

#### Biofilm Formation

*In vitro* biofilm formation was investigated as described by Laverede Gomez et al. (35) with slight modification. Bacterial cells from exponential growth phase were incubated for 48 h at 37°C in 96-well microliter plates. The planktonic culture was removed and the biofilm (placed on the bottom of the well) was washed with PBS and stained with crystal violet solution (Roth, Karlsruhe, Germany). The absorbance at OD_595nm_of the 99.8% ethanol used to destain the biofilm was measured. The biofilm formation was determined as OD600_nm_/OD595_nm_ (ratio between culture and destaining solution absorbance).

#### Determination of Antimicrobial Susceptibility

The three strains were tested for susceptibility to 18 antibiotics, including different antibiotic classes such as aminoglycosides and glycopeptides, using the broth microdilution method according to DIN58940 (46). The following antibiotics were used: penicillin, ampicillin, gentamycin, streptomycin, vancomycin, teicoplanin, daptomycin, clindamycin, erythromycin, ciprofloxacin, moxifloxacin, tetracycline, tigecycline, rifampicin, linezolid, mupirocin, chloramphenicol, and trimethoprim/sulfamethoxazole. The test was performed according to EUCAST breakpoints definitions (v 8.1) or to ECOFF (epidemiological cut-off) values when no breakpoints were defined (www.eucast.org). As a quality control, also the *E. faecalis* ATCC29212 was tested in this experiment, as recommended by EUCAST guidelines.

## Results

The extracellular and intracellular proteomic profiles of the food-isolate *Enterococcus faecalis* D27 (from now referred to as D27) were compared to the profiles of the probiotic *E. faecalis* Symbioflor 1 (from now referred to as Symb1) and the pathogenic *E. faecalis* UW3114 (from now referred to as UW3114), in order to assess the potential risks and benefits associated to the not well-characterized *E. faecalis* D27. Phenotypic aspects connected to virulence/pathogenesis were also investigated.

### Gel-free Proteomic Analyses

In order to highlight the proteins directly or partially related to pathogenicity possible present in each of the three investigated strains, the (NCBI) database of *E. faecalis* OG1RF was used for protein identification. Moreover, to identify proteins encoded by the pathogenicity island (PAI) of UW3114 the database also contained the sequences of the PAI of strain MH594, since many of the proteins encoded by this PAI were also found on the PAI of UW3114 (35). Only proteins present in at least two of the three biological replicates, were considered for comparative analysis. The proteomic pattern obtained for the food strain D27, was compared to those of the probiotic strain Symb1 and the clinical isolate UW3114 separately. This approach allowed to establish: (i) which proteins are expressed by both matched bacteria but in different amounts and (ii) which proteins belong to the so called on/off proteins, that are synthesized by one strain but not by the other.

**Figure 1:**
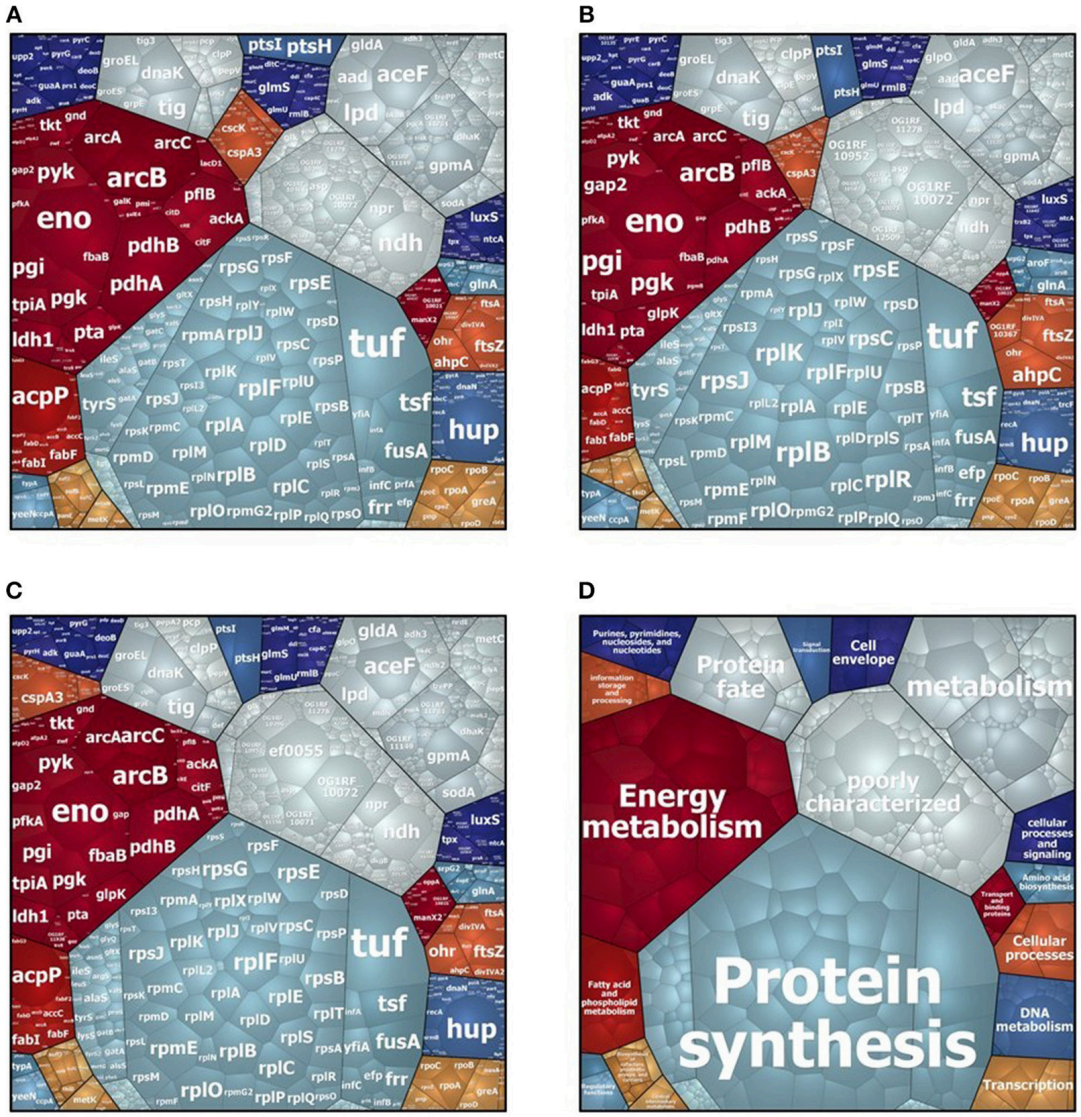
Voronoi treemap visualization of cytosolic proteins of *E. faecalis* D27 **(A)**, *E. faecalis* Symbioflor 1 **(B)** and *E. faecalis* UW3114 **(C)**. Each protein is represented by a small cell, proteins are clustered according to their functional classification, cell-size correlates with protein abundance. **(D)** Represents functional organization of proteins.

In the intracellular compartment of D27, Symb1, and UW3114 we identified 889, 883, and 860 proteins, respectively (Tables S1, S2). The classification of intracellular proteins in functional groups reveals similar profiles among the three strains (Figure 1), including proteins involved in: (I) protein synthesis, (II) energy metabolism (III) metabolism (metabolite transport and energy conversion), (IV) protein fate and (IV) poorly characterized proteins. The most striking difference is the presence of the high-abundant protein Gls24-like protein Ef0055 in strain UW3114, located on the pathogenicity island. In the secretome we identified 440, 370 and 253 proteins for D27, Symb1, and UW3114, respectively (Tables S3, S4). Only proteins with extracellular, cell wall, cell membrane or unknown sub-localization in the localization prediction by pSortb (version 3.0.2) were considered for the discussion.

#### Comparison Food/Probiotic

**Figure 2:**
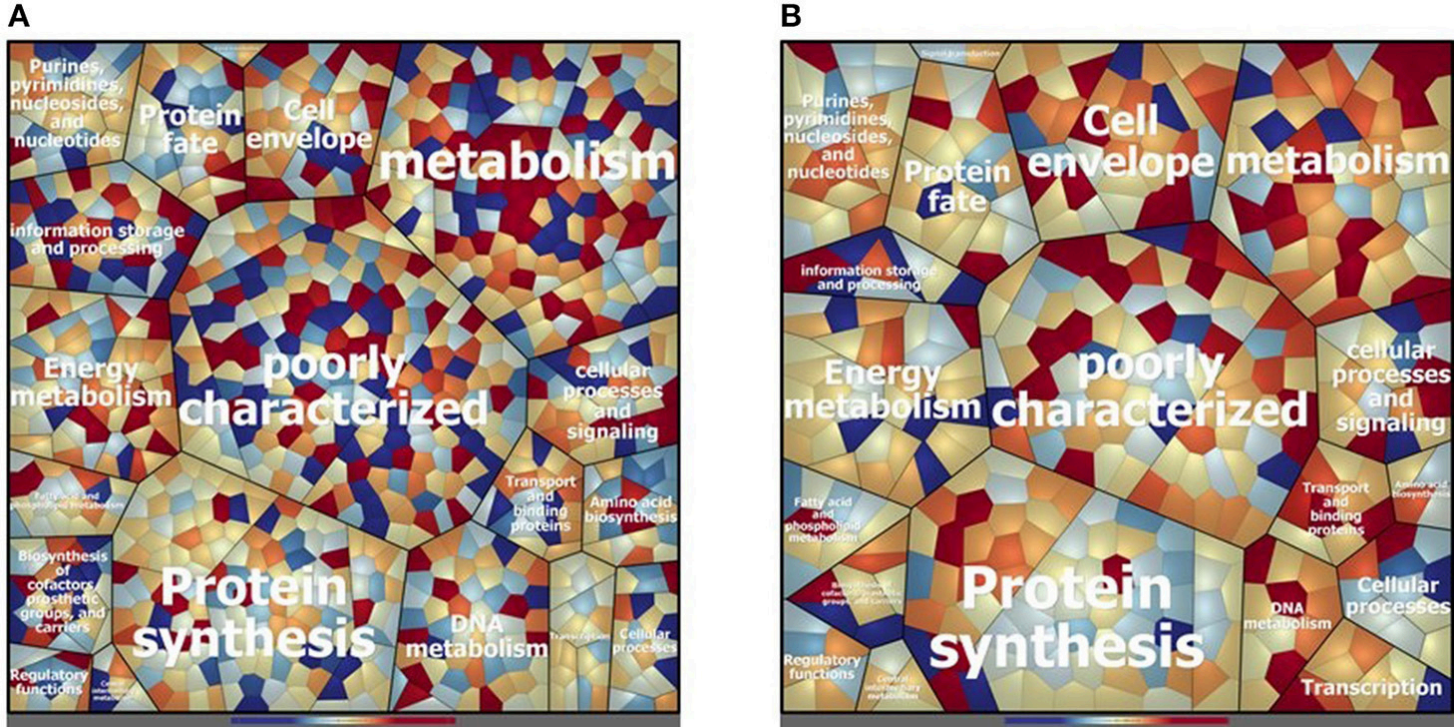
Voronoi treemap visualization depicting the different protein pattern of *E. faecalis* D27 and *E. faecalis* Symbioflor 1 in the cytosolic **(A)** and extracellular **(B)** compartment. The proteins are grouped according to their predicted functional classification. Variation of relative protein amounts are indicated by color: red indicates higher amount in D27, blue higher abundance in Symbioflor 1.

Concerning the cytosolic compartment, 794 shared proteins (88 % of the identified proteins), between D27 and Symb1, were identified by mass spectrometry analysis (Table S1). Figure 2A shows a general overview on the different proteins amount in D27 in comparison to Symb1 (color variation in a scale from red, abundant in D27 to blue, abundant in Symb1). Quantitative differences were detected, especially for proteins involved in metabolism, cell envelope, information storage and processing. Interestingly, the “poorly characterized” protein area comprises a high number of significant differentially expressed proteins (Figure 2A).

The proteins that are more abundant in D27 as compared to Symb1 are 37 and, among them the conserved protein UCP028846 (18.27 fold change - OG1RF_11419), tagatose-biphosphate aldolase (11.95 fold change - OG1RF_10434), 1-phosphofructokinase (7.70 fold change - OG1RF_10431), exonuclease SbcC (6.88 fold change - OG1RF_12058) and glycosil hydrolase (6.05 fold change - OG1RF_12425) show the highest fold change values. For what concern the 95 proteins present in D27 and absent in Symb1, are worth mentioning the hemolysin (OG1RF_10438) and the penicillin-binding protein 1B (OG1RF_11450) (Table S1).

D27 and Symb1 share 344 extracellular proteins that represent about the 90% of the identified proteins. The Voronoi treemap (Figure 2B) provides an overview of the differentially expressed proteins between the two strains according to the quantitative data [based on the normalized spectrum abundance factors (NSAF)]. As suggested by the graphic visualization, most of the common proteins show a comparable level in two strains considered. Forty proteins are more abundant in D27 as compared Symb1, showing fold changes ranging between 5.63 and 2.05, most belonging to membrane transporters and cell-wall biosynthetic enzymes. The proteins present in D27 and absent in Symb1 are 95, where the gelatinase (OG1RF_11526) is the most interesting identified protein (Table S3).

#### Comparison Food/Pathogen

**Figure 3:**
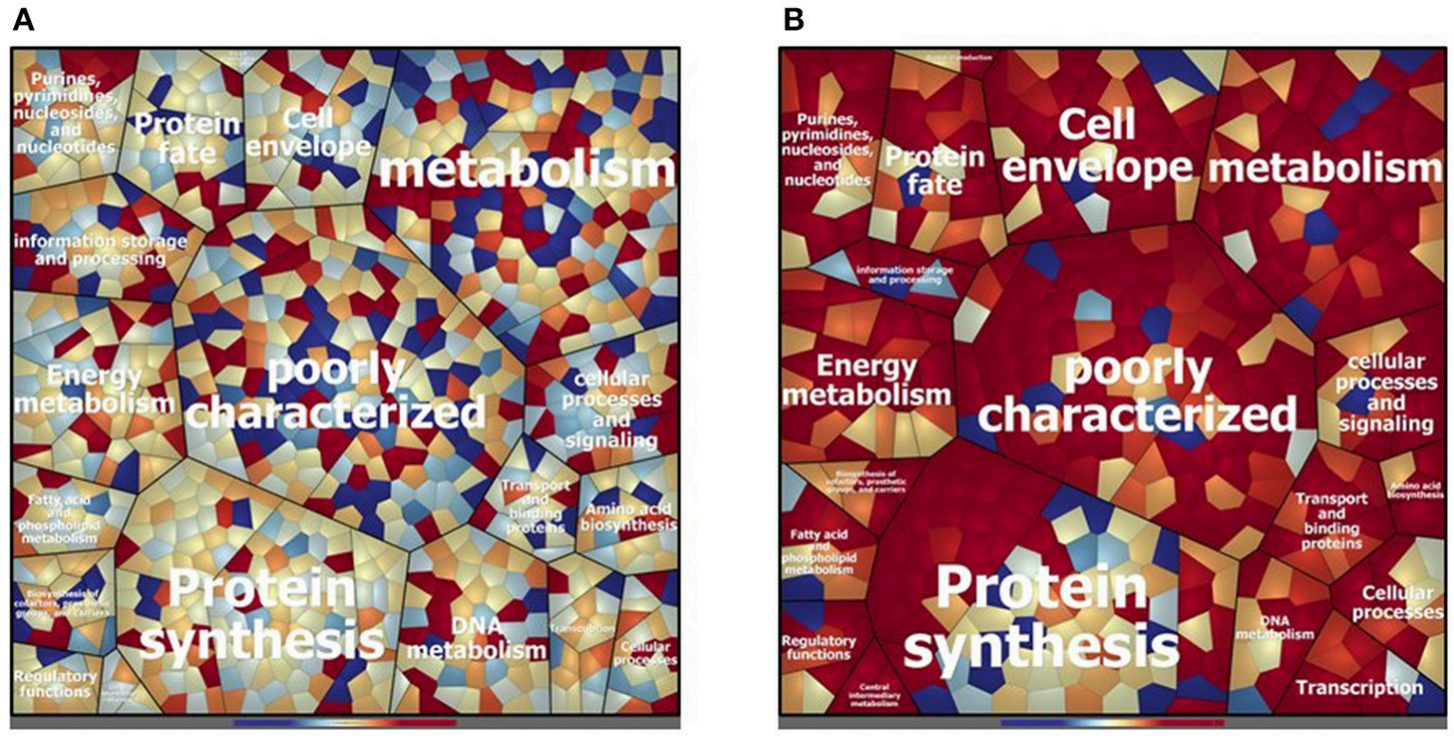
Voronoi treemap visualization depicting the different protein pattern of *E. faecalis* D27 and *E. faecalis* UW3114 in the cytosolic **(A)** and extracellular **(B)** compartment. The proteins are grouped according to their predicted functional classification. Variation of relative protein amounts are indicated by color: red indicates higher amount in D27, blue higher abundance in UW3114.

The cytosolic identified proteins shared by the dairy-isolate D27 and the clinical isolate UW3114 are 769, corresponding to the 87% of the total identified proteins (Table S2). As noticed previously, comparing D27 and Symb1, most of the proteins were assigned to the functional groups of metabolism-involved, cell envelope, information storage/processing and poorly characterized proteins (Figure 3A). The proteins more abundant in D27, as compared to UW3114, are 43 (Table S2) and among them the aldehyde-alcohol dehydrogenase (9.47 fold change - OG1RF_10627), phosphoglycerate mutase (5.50 fold change - OG1RF_12264), formate acetyltransferase (5.30 fold change - OG1RF_11329), 2-dehydropantoate 2-reductase (5.20 fold change - OG1RF_11367), uracil phosphoribosyltransferase (5.14 fold change - OG1RF_11432) show the highest fold change values. For what concern the 120 proteins present in D27 and absent in UW3114, are worth mentioning the hemolysin A (OG1RF_10716) and the β-lactamase (OG1RF_11969).

In the extracellular compartment of the D27 and UW3114, 226 proteins expressed by both strains were found (Table S4). The Voronoi treemap (Figure 3B) outlines how most of the proteins (red cells) are more abundant in the food isolate compared to the clinical strain. In particular, 116 proteins were more abundant in D27 compared to UW3114, showing fold change ranging between 21.06 and 2.01 (Table S4). Besides membrane transporters and cell-wall biosynthetic enzymes are worth mentioning a penicillin-binding protein C (13.75 fold-change - OG1RF_10724) and a β-lactamase (6.98 fold-change - OG1RF_11219). The different protein profiles between strains, indicated by the high fold change variation of the common proteins, is also supported by the presence of 212 proteins only identified in D27 and absent in UW3114 (Table S4). Among these, the most interesting are: choloylglycine hydrolase (AAM75246.1_39), efflux transporter (RND family - OG1RF_10301), PFL4705 family integrating conjugative element protein (OG1RF_12168) and chitin-binding protein (OG1RF_12499) (Table S4).

### Phenotypic Tests

#### Evaluation of Enzymatic Activities Related to Pathogenicity

**Figure 4:**
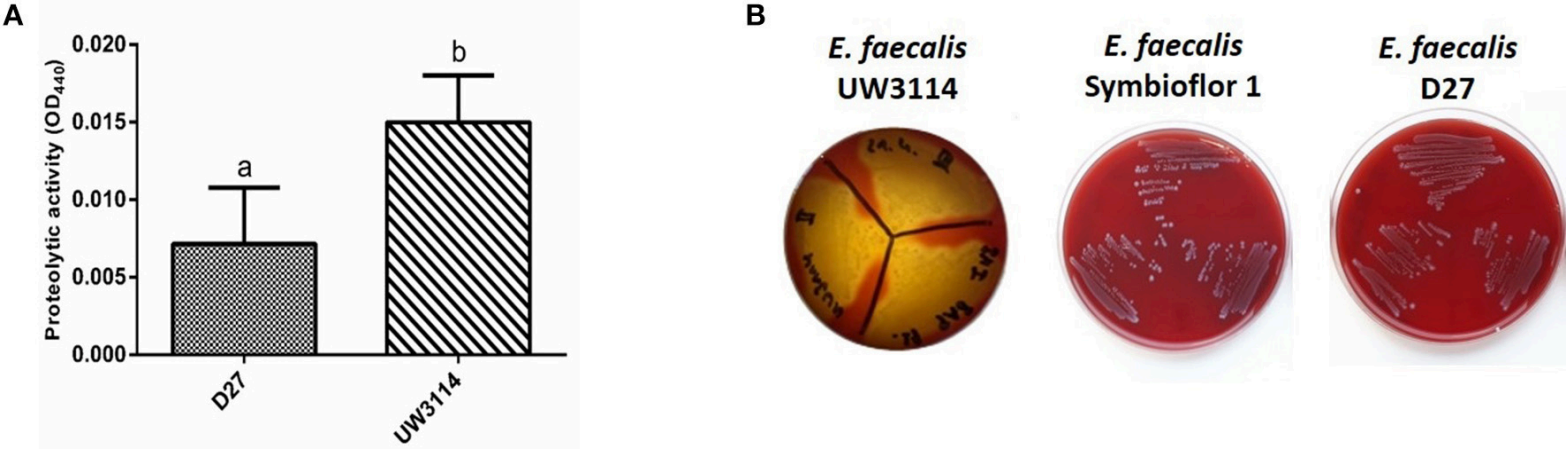
**(A)** proteolytic activity, measured as optical density (OD) at 440 nm, in *E. faecalis* D27 and *E. faecalis* UW3114; **(B)** hemolytic activity of *E. faecalis* UW3114, Symbioflor 1 and D27 tested on horse blood agar plates. Only *E. faecalis* UW3114 shows β-hemolytic activity.

Protease and hemolysis were evaluated in the three strains in study, being the most common enzymatic activities associated to virulence, and the results are reported in Figure 4. As shown in Figure 4A, the strain UW3114 displays a 2-fold more intense protease activity than the food-isolate D27. As respect to the probiotic, no comparison can be done since for the latter measured absorbance values were negative. Concerning hemolysis, only the clinical isolate UW3114 shows hemolytic activity, against horse blood cells, where a β-hemolysis pattern (formation of clear halos) was observed (Figure 4B). Concerning gelatinase all the three strains were negative (data not shown).

#### Biofilm Formation

**Figure 5:**
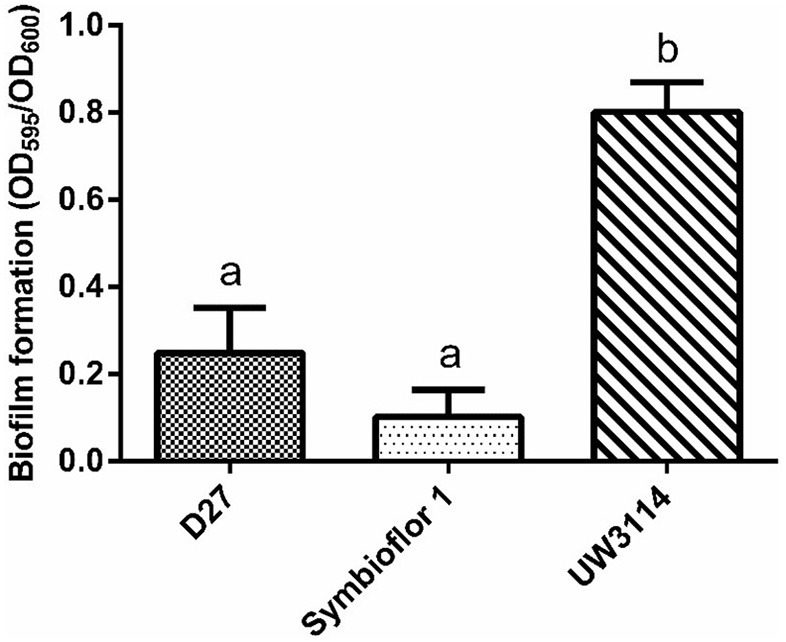
biofilm quantification after crystal violet staining. The amount of biofilm produced by *E. faecalis* D27, *E. faecalis* Symbioflor 1, and *E. faecalis* UW3114 is proportional to the ratio between the crystal violet OD value (595) and culture OD value (600).

The ability to produce extracellular matrix was evaluated by growing bacteria on an abiotic solid surface, to allow biofilm formation. Although the three strains were able to secrete extracellular polymeric substances, biofilm quantification (using crystal violet staining) demonstrated that for the clinical strain biofilm is 8-fold more abundant than for Symb1 and 4-fold more than for D27 (Figure 5).

#### Antibiotic-Resistance Profiles

To get general insights in the antibiotics susceptibilities and resistances of the analyzed *E. faecalis* strains, 18 different antibiotics were tested and the results reported in Table 1. All the three isolates showed species-specific resistance to mupirocin (MUP) and also to clindamycin (CLI). These results were expected in enterococci, and confirmed by the test on the reference strain *E. faecalis* ATCC29212. None of the isolates showed resistance to glycopeptide compounds (vancomycin and teicoplanin) or to penicillin antibiotics (penicillin and ampicillin). Both the probiotic and the dairy-isolated strain D27 showed to be susceptible to 16 analyzed antibiotics. Conversely, the clinical isolate UW3114 was found to be resistant to 8 antibiotics, among which erythromycin. Erythromycin-resistance is ascribable to the presence of the PAI, as described before by Laverde Gomez (35).

**Table 1:** Antibiotic susceptibility (green) and resistance (red) of the three tested strains of *E. faecalis*, D27, UW3114, and Symbioflor 1, and the quality control strain *E. faecalis* ATCC29212, as recommended by EUCAST guidelines.

	**D27**	**UW3114**	**Symbioflor1**	**ATCC29212**
PEN	2	8	4	2
AMP	2	4	4	2
GEN	≤ 64	512	≤ 64	≤ 64
STR	≤ 128	2,048	≤ 128	≤ 128
VAN	2	2	4	4
TPL	≤ 1	≤ 1	≤ 1	≤ 1
DAP	≤ 2	≤ 2	≤ 2	≤ 2
CLI	>8	>8	8	8
ERY	≤ 1	>16	≤ 1	≤ 1
CIP	≤ 2	32	≤ 2	≤ 2
MFL	≤ 0.125	>2	≤ 0.125	0.25
LNZ	≤ 2	≤ 2	≤ 2	≤ 2
TET	≤ 0.5	>16	≤ 0.5	>16
TGC	≤ 0.063	≤ 0.063	≤ 0.063	≤ 0.063
RAM	2	≤ 1	4	≤ 1
SXT	≤ 0.032	≤ 0.032	≤ 0.032	≤ 0.032
CMP	8	8	8	8
MUP	>32	>32	>32	>32

*PEN, penicillin; AMP, ampicillin; GEN, gentamycin; STR, streptomycin; VAN, vancomycin; TPL, teicoplanin; DAP, daptomycin; CLI, clindamycin; ERY, erythromycin; CIP, ciprofloxacin; MFL, moxifloxacin; LNZ, linezolid; TET, tetracycline; TGC, tigecycline; RAM, rifampicin; SXT, trimethoprim/sulfamethoxazole; CMP, chloramphenicol; MUP, mupirocin*.

## Discussion

Establishing the safety of a food-isolated strain is of primary importance especially when the controversial species *E. faecalis* is concerned. Actually, enterococci are not generally regarded as safe (GRAS) by e.g., the FDA. Proteomics is a valuable strategy to detect virulence characters. Furthermore, comparison with clearly recognized pathogenic and probiotic bacteria is an added value to highlight differences useful to classify the food-isolated strains as safe.

The *E. faecalis* in study D27 was compared to a clinical isolate UW3114 and to a commercialized probiotic Symb1. Several proteins display a significant fold-change as compared to the other two enterococci or are exclusively present in the food isolate. Of these, some are worth discussion because of their implication in pathogenicity, antibiotic resistance, gene transfer and possible human-host interaction.

### Proteins Involved (or Possibly Involved) in Pathogenicity

Several proteins connected with pathogenic traits are expressed by the dairy-isolate D27. The protein named “secreted antigen” was found to be a serine protease by database search (BLAST comparison with *E. faecalis* strains). The serine protease HtrA (OG1RF_12305) was expressed by both D27 and UW3114, however it was 2-fold more abundant in the clinical isolate. In the secretome, a different serine protease SprE (OG1RF_11525) was detected only in the hospital-isolated strain UW3114. Although D27 does not express SprE, another serine protease with pathogenic implications was found, the S41A family carboxy-terminal peptidase (OG1RF_11392). It has been reported that, in a murine sepsis model, disease caused by a S41-deficient mutant of *S. aureus* was less severe than that caused by the WT strain; this result demonstrates the role of these proteins in Gram-positive pathogenicity (47). A further proteolytic enzyme found in both D27 and UW3114 secretome is gelatinase (OG1RF_11526), one among the most important virulence factors in *E. faecalis*. The presence of genes for gelatinase E (GelE) and serine protease V8 (SprE) is usually considered a marker to identify potential pathogenic strains (48). However, it was already observed that a cheese-isolated *E. faecalis* strain does not show gelatinase or protease activity when submitted to phenotypic tests, even if possessing the abovementioned genes (16). This phenomenon was also observed in strains D27 and UW3114, where the presence of gelatinase enzyme does not support gelatinase activity. Two different types of hemolysins were present: hemolysin (OG1RF_10438) and hemolysin A (OG1RF_10716*)*. D27 expresses both enzymes, whereas both the probiotic and the clinical strains express only one of them. However, the clinical isolate is the only strain showing β-hemolysis *in vitro*, as expected (Figure 4B). Actually, it is long time established that the presence of the *cyl* operon is indispensable but not sufficient for the hemolytic activity, since different enzymes are involved and a proteolytic cleavage is required for protein activation (49). The influence of environmental factors on gene expression is a well-known phenomenon. For Enterococcus, the effect of multiple external factors on pathogenic character expression has been described since 1998 (50).

Finally, a LemA family protein (OG1RF_10353) was found in D27 and UW3114, with less than 2-fold change in abundance. This protein family was well characterized in the *Pseudomonas* genus, where it was identified as a transmembrane histidine protein kinase (HPK) sensor-regulators, involved in lesion formation in the host (51).

### Proteins Involved in Antibiotic Resistance

As a general rule, it has to be highlighted that in Enterococci, antibiotic resistance is based upon three main mechanisms: (a) intrinsic or natural resistance (b) acquired resistance and (c) tolerance. The intrinsic resistance is a genus-related feature and it is generally linked to insensitivity of the bacterial cell to the antibiotic molecules either through absence of the uptake mechanism (5), or to a modification of the antibiotic targets such as the PBPs (penicillin binding proteins) (52) or to efflux systems (53). Discovered in the 1980 (54) and referred to also as “intrinsic insensitivity” it is the main cause of the difficulty of treating enterococcal infections (55). The acquired resistance, on the contrary, is often based upon production of enzymes modifying or degrading the antibacterial molecule and it is transmissible by genetic recombination. Hence, this last mechanism is more dangerous since it can allow spread of the resistance character among strains (5).

Two classes of proteins were found that could confer antibiotic resistance to the *E. faecalis* strains under investigation: the true resistance marker β-lactamase and the penicillin-binding proteins (PBPs) that can be involved in resistance if a mutation in their sequence occurs. The three strains show to possess a heterogeneous set of these proteins.

As far as the membrane-located PBPs are concerned, their relative abundance evaluation has the limitation of a low reliability, because the extraction procedure could not have been homogeneous in the three samples analyzed. PBPs (e.g., PBP2B, PBP3, PBP4, PBP5) are involved in the peptidoglycan formation. However, it was observed that the presence of a high molecular weight variant of PBP5 decreased susceptibility of *E. faecium* to ampicillin and other β-lactams (56, 57). In *E. faecalis*, resistance to both imipenem and ampicillin due to amino acid mutation (520 and 605 residues) in the PBP4 was observed (58). In the cytosolic proteome of the studied strains, the PBP1A (OG1RF_10925) seems 2.5-fold more abundant in D27 compared to Symb1; the PBP1B (OG1RF_11450) is exclusively expressed by D27; the PBPC (OG1RF_10724) was found in large amount in D27, compared to the other two strains. Furthermore, extracellular proteins identification in the food strain confirmed the presence of PBPs, with some differences respect the cytosolic compartment. PBP1A that in the cytosol was found more abundant in D27 compared to Symb1, in the extracellular compartment appears to be more abundant in the probiotic strain. Therefore, it seems that this abundance is rather due to compartmentalization than to higher gene expression.

Three different β-lactamases were found, both in the intra- and extra-cellular proteomes. Two of them are metallo-hydrolases, one exclusively expressed in Symb1 (OG1RF_10969) and the other (OG1RF_11863) 2.5-fold more abundant in the clinical isolate compared to the food-isolate; the last is a serine-hydrolase (OG1RF_11969) exclusively expressed in D27. The antibiotic inactivation based on hydrolytic activity is mostly effective when applied to β-lactam antibiotics. In particular, there are two main molecular strategies employed by β-lactamases to hydrolytically cleave the β-lactam ring of penicillins and cephalosporins: through the action of an active site Ser-nucleophile, or through activation of water via a Zn^2+^ center (59). As reported in the literature, the proteins described above are responsible for insensitivity to β-lactam antibiotics in enterococci (60). Furthermore, this type of resistance seems to be transmissible (5).

The identification of a high variety of proteins, possibly involved in resistance, found in the proteomes of the three strains in study, suggests that resistance events could occur also in the probiotic and the dairy-isolate that share a similar antibiotitype. Actually, probiotics were already described as harboring resistance traits (61). However, in spite of these protein profiles, the antibiotic susceptibility testing revealed a general sensitivity pattern toward beta-lactams in all the three strains considered (Table 1). As far as the PBPs are concerned, it is possible that they are active and not mutated forms as those described in *E faecalis* by Ono (58). Regarding beta lactamases, it is possible to hypothesize that the expression of these proteins is a condition necessary but not sufficient to engender resistance, suggesting that other factors can play a role in the occurrence of resistant phenotypes.

As far as the food-borne *Enterococcus* D27 is concerned, an efflux transporter (RND family - OG1RF_10301) has also been found. Efflux pumps, responsible either of multidrug resistance mechanisms or of single antibiotic extrusion, have been described in LAB as well (62). In the strain D27 this efflux system could be involved in the clindamycin resistance pattern observed (Table 1), whereas the resistance to mupirocin observed seems to be due to other mechanisms as it has long been established (63). On the other hand, both these resistances are present in all the three strain considered, suggesting that they can be based upon an intrinsic resistance mechanism as frequently described for Enterococci (55).

### Proteins Involved in Horizontal Gene Transfer

The PFL4705 family integrating conjugative element protein (OG1RF_12168) was found both in the food-isolate D27 and in the probiotic strain Symb1, with comparable expression levels. Often, virulence determinants are acquired as mobile elements during gene transfer processes also mediated by this protein and these genetic exchanges are frequent between *E. faecalis* strains (35, 64).The second interesting protein more expressed by D27, is the pheromone cAM373 (OG1RF_11130). It was characterized also in *S. aureus* and corresponds to a heptapeptide (AIFILAS) located within the C-termini of the signal sequences of putative pre-lipoproteins. This hydrophobic, linear peptide molecule acts as signals that facilitate the conjugative transfer of a specific category of plasmids referred to as pheromone-responsive plasmids (65). This specific type of inducible plasmid represents an important mechanism for dissemination of antibiotic resistance and virulence among *Enterococcus* strains (66). These findings also suggest that the food ecosystem is a suitable environment for favoring cell-to-cell communication.

In the extracellular fraction, a second pheromone, cAD1 (OG1RF_12509), more expressed by the dairy-isolate, was found. In *E. faecalis*, this octapeptide sex pheromone was identified as responsible of an induced mating response by donors carrying the hemolysin plasmid pAD1 or related elements (67). This result, although expected for the clinical isolate is surprising for the food strain that generally do not need to acquire hemolysins in its ecological niche.

### Proteins Involved in Human Host/Environment Interactions

The von Willebrand factor (vWF) type A domain protein (OG1RF_10869) is an example of potential pathogenicity-associated protein more expressed in the secretome by the food-isolate strain compared to the probiotic and clinical isolates (fold-change 2.21 and 2.16, respectively). vWF is a huge multimeric protein, well-characterized in humans, where it triggers platelet adhesion in areas of vascular damage. In particular, the domain A1 mediates platelet adhesion under flow in areas of vessel injury through the platelet glycoprotein Ibα (GPIbα) (68). Most prokaryotic vWF domains have not been investigated in detail, however, Konto-Ghiorghi et al. (69) showed that the GBS PilA tip pilin vWF domain was important for pilus-mediated bacterial adhesion to human alveolar and intestinal epithelial cells *in vitro*. The last newsworthy evidence is the variety of lipoproteins produced by the dairy-isolate. Even if the function and the localization is still unknown for most of them it is well-recognized that lipoproteins in Gram-positive bacteria represent about the 25% of the surface associated proteins, which could play a major role in bacterial virulence processes (70).

Among up-regulated proteins in D27, choloylglycine hydrolase (AAM75246.1_39) represents an interesting enzyme since it catalyzes the initial “gateway” reaction in the bacterial metabolism of CBAs (conjugated bile acids). The reaction consists in a deconjugation of CBAs to liberate free primary bile acids (BAs; cholic acid or chenodeoxycholic acid) and amino acids (71). CBAs have been suggested to repress bacterial growth in the small intestine by means of direct antimicrobial effects, up-regulation of host mucosal defenses, or synergistic action of both mechanisms (72). The expression of this enzyme by the food-isolate can increase the ability of the strain to survive to CBAs action in the gut, thus enhancing the probiotic potential of D27. This is partly expected, because Enterococci generally display a high degree of resistance to bile salts (73). Considering that, all the three strains share the presence of several proteins involved in mucosa and mucus adhesion (e.g., enolase, EFTu, EFTs, DnaK, Clp, GroEL, chitin-binding protein) (74, 75), it is possible to hypothesize that the foodborne D27 can also share the same attitude to persist in the human host by adhering to the gut mucosa. However, D27 ability to form biofilm, although higher than Symb1, is four-fold lower than the one observed in the pathogenic UW3114.

## Conclusion

The results obtained in the present investigation comparing a food-isolated *E. faecalis* D27 with a patented probiotic and a pathogenic isolate, underline that there is the need of a detailed typing of strains, either employed in food fermentation or foodborne contaminants, to avoid risks of virulence dissemination.

The probiotic strain *E. faecalis* Symbioflor 1, although bearing some resistance traits, possess several proteins that support its role as a probiotic, namely involved in stress response, that are of primary importance both for the shelf-life of the probiotic preparation and for bacterial survival in the human gastro-intestinal tract. The clinical isolate *E. faecalis* UW3114 was the only strain synthesizing some of the ascertained virulence determinants such as the serine protease SprE and displaying the highest levels of antibiotic resistance included aminoglycosides. All these results were expected.

As far as the foodborne isolate is concerned, *E. faecalis* D27 besides showing a high metabolic activity (confirmed by the up-regulation of several proteins involved in hydrolysis, energy metabolism, and nutrient transport) also displays some proteins whose function could be hazardous. Among these, antibiotic resistance factors (although not all expressed in the tested conditions) and horizontal gene transfer involved proteins (PFL4705 family integrating conjugative element), are a clear evidence that recombination events (both in food and in the GIT) can cause antibiotic resistance spread. Other possible virulence factors are the vWF type A domain protein, and some proteases involved in pathogenicity, like the serine protease HtrA, the S41A family carboxy-terminal peptidase and LemA. On the other hand, the presence in D27 of a high number of proteolytic enzymes is probably linked to the advantage of possessing proteases in a food matrix like cheese that is very rich in proteins. It is worth to highlight that single approaches for typing bacteria to find pathogenicity factors all have the limitation of considering characters (genes or proteins) that not always are disclosed.

Considering the heterogeneous habitat in which food microorganisms live, modifications of their proteomic patterns can also occur both by interaction with other bacteria (genetic exchanges) and during contact with the host. The results obtained in the present investigation are far to be exhaustive since new genes and proteins involved in pathogenesis in different *E. faecalis* strains are repeatedly reported in the literature. However, taken together, these data demonstrate the importance of carefully and periodically characterize food-isolated enterococcal strains to ascertain their safety before employing them for human consumption. In this context, in parallel to genetic analysis, complementing gel-free proteomics (especially the analyses concerning secreted “marker-factors”) and phenotypic tests proved to be a valuable tool to assess these features.

## Author Contributions

All authors listed have made a substantial, direct and intellectual contribution to the work, and approved it for publication.

### Conflict of Interest Statement

The authors declare that the research was conducted in the absence of any commercial or financial relationships that could be construed as a potential conflict of interest.

## Supplementary Material

Table S1 The table includes the cytosolic proteins identified in at least two of the three biological replicates of *E. faecalis* D27 and *E. faecalis* Symbioflor 1 and the proteins found only in one *E. faecalis* strain and absent (off) in the other strain. The fold-change is given by the ratio between the average of the protein abundance (fmol/ng) in *E. faecalis* D27 and the average of the protein abundance (fmol/ng) in *E. faecalis* Symbioflor 1. Accession, protein name, description, localization (obtained by pSortb), main role and subrole are reported to give information on the protein function. For each protein, the abundance (fmol/ng) for all the biological replicates, as well as the average of the abundance are reported for both *E. faecalis* D27 and *E. faecalis* Symbioflor 1. The Repcount refers to the number of technical replicates in which the protein was identified.

Table S2 The table includes the cytosolic proteins identified in at least two of the three biological replicates of *E. faecalis* D27 and *E. faecalis* UW3114 and the proteins found only in one *E. faecalis* strain and absent (off) in the other strain. The fold-change is given by the ratio between the average of the relative abundance (fmol/ng) in *E. faecalis* D27 and the average of the relative abundance (fmol/ng) in *E. faecalis* UW3114. Accession, protein name, description, localization (obtained by pSortb), main role and subrole are reported to give information on the protein function. For each protein, the abundance (fmol/ng) for all the biological replicates, as well as the average of the abundance are reported for both *E. faecalis* D27 and *E. faecalis* UW3114. The Repcount refers to the number of technical replicates in which the protein was identified.

Table S3 The table includes the secreted proteins identified in at least two of the three biological replicates of *E. faecalis* D27 and *E. faecalis* Symbioflor 1 and the proteins found only in one *E. faecalis* strain and absent (off) in the other strain. The fold-change is given by the ratio between the average of the abundance [normalized spectral abundance factor (NSAF)] in *E. faecalis* D27 and the average of the abundance (NSAF) in *E. faecalis* Symbioflor 1. Accession, protein name, description, localization (obtained by pSortb), main role and subrole are reported to give information on the protein function. For each protein, the abundance (NSAF) for all the biological replicates are reported for both *E. faecalis* D27 and *E. faecalis* Symbioflor 1.

Table S4 The table includes the secreted proteins identified in at least two of the three biological replicates of *E. faecalis* D27 and *E. faecalis* UW3114 and the proteins found only in one of *E. faecalis* strain and absent (off) in the other strain. The fold-change is given by the ratio between the average of the abundance [normalized spectral abundance factor (NSAF)] in *E. faecalis* D27 and the average of the abundance (NSAF) in *E. faecalis* UW3114. Accession, protein name, description, localization (obtained by pSortb), main role and subrole are reported to give information on the protein function. For each protein, the abundance (NSAF) for all the biological replicates are reported for both *E. faecalis* D27 and *E. faecalis* UW3114.

## References

[1] Arias CA Murray BE Emergence and management of drug-resistant enterococcal infections, microbial drug resistance, future medicine. Exp Rev Anti Infect Ther. (2008) 6:637–55. 10.1586/14787210.6.5.637 18847403

[2] Schaberg DR Culver DH Gaynes RP. Major trends in the microbial etiology of nosocomial infection. Am J Med. (1991) 91:S72–5. 10.1016/0002-9343(91)90346-Y 1928195

[3] Mathur S Singh R. Antibiotic resistance in food lactic acid bacteria—a review. Int J Food Microbiol. (2005) 105:281–95. 10.1016/J.IJFOODMICRO.2005.03.008 16289406

[4] Nam HM Lim SK Moon JS Kang HM Kim JM Jang KC. Antimicrobial resistance of enterococci isolated from mastitic bovine milk samples in Korea. Zoonoses Public Health. (2010) 57: e59–64. 10.1111/j.1863-2378.2009.01307.x 20042062

[5] Argyri AA Zoumpopoulou G Karatzas KAG Tsakalidou E Nychas GJE Panagou EZ. Selection of potential probiotic lactic acid bacteria from fermented olives by *in vitro* tests. Food Microbiol. (2013) 33:282–91. 10.1016/j.fm.2012.10.005 23200662

[6] Miller WR Munita JM Arias CA. Mechanisms of antibiotic resistance in enterococci. Expert Rev Anti Infect Ther. (2014) 12:1221–36. 10.1586/14787210.2014.956092 25199988 PMC4433168

[7] Hegstad K Mikalsen T Coque TM Werner G Sundsfjord A. Mobile genetic elements and their contribution to the emergence of antimicrobial resistant *Enterococcus faecalis* and *Enterococcus faecium*. Clin Microbiol Infect. (2010) 16:541–54. 10.1111/j.1469-0691.2010.03226.x 20569265

[8] Levy SB Bonnie M. Antibacterial resistance worldwide: causes, challenges and responses. Nat Med. (2004) 10:S122–9. 10.1038/nm1145 15577930

[9] Salyers AA Shoemaker NB. Resistance gene transfer in anaerobes: new insights, new problems. Clin Infect Dis. (1996) 23 (Suppl. 1):S36–43. 10.1017/s0950268812000635 8953105

[10] Eaton T Gasson M. Molecular screening of enterococcus virulance determinants and potential for genetic exchange between food and medical isolates. Appl Environ Microbiol. (2001) 67:1628–35. 10.1128/AEM.67.4.1628 11282615 PMC92779

[11] Mannu L Paba A Daga E Comunian R Zanetti S Duprè I. Comparison of the incidence of virulence determinants and antibiotic resistance between *Enterococcus faecium* strains of dairy, animal and clinical origin. Int. J. Food Microbiol. (2003) 88:291–304. 10.1016/S0168-1605(03)00191-0 14597001

[12] Shankar N Lockatell CV Baghdayan AS Drachenberg C Gilmore MS Johnson DE. Role of Enterococcus faecalis surface protein esp in the pathogenesis of ascending urinary tract Infection. (2001) 69:4366–72. 10.1128/IAI.69.7.4366 11401975 PMC98508

[13] Daw K Baghdayan AS Awasthi S Shankar N. Biofilm and planktonic Enterococcus faecalis elicit different responses from host phagocytes *in vitro*. FEMS Immunol Med Microbiol. (2012) 65:270–82. 10.1111/j.1574-695X.2012.00944.x 22333034 PMC3366019

[14] Klein G. Taxonomy, ecology and antibiotic resistance of enterococci from food and the gastro-intestinal tract. Int J Food Microbiol. (2003) 88:123–31. 10.1016/S0168-1605(03)00175-2 14596985

[15] Kayaoglu G Ömürlü H Akca G Gürel M Gençay Ö Sorkun K. Antibacterial activity of propolis versus conventional endodontic disinfectants against *Enterococcus faecalis* in infected dentinal tubules. J Endod. (2011) 37:376–381. 10.1016/J.JOEN.2010.11.024 21329825

[16] Pessione A Lamberti C Cocolin L Campolongo S Grunau A Giubergia S. Different protein expression profiles in cheese and clinical isolates of *Enterococcus faecalis* revealed by proteomic analysis. Proteomics. (2012) 12:431–47. 10.1002/pmic.201100468 22213736

[17] Abriouel H Omar N Ben M López RL Grande MJ Martínez-Viedma P. Comparative analysis of genetic diversity and incidence of virulence factors and antibiotic resistance among enterococcal populations from raw fruit and vegetable foods, water and soil, and clinical samples. Int J Food Microbiol. (2008) 123:38–49. 10.1016/J.IJFOODMICRO.2007.11.067 18180067

[18] Vachee A Drider D Silvain A Al Atya AK Ravallec R Drider-Hadiouche K. Probiotic potential of *Enterococcus faecalis* strains isolated from meconium. Front Microbiol. (2015) 6:227. 10.3389/fmicb.2015.00227 25883590 PMC4382979

[19] Shepard BD Gilmore MS. Antibiotic-resistant enterococci: the mechanisms and dynamics of drug introduction and resistance. Microbes Infect. (2002) 4:215–24. 10.1016/S1286-4579(01)01530-1 11880055

[20] Tyson GH Nyirabahizi E Crarey E Kabera C Lam C Rice-Trujillo C Prevalence and antimicrobial resistance of Enterococci isolated from retail meats in the United States, 2002-2014. Appl Environ Microbiol. (2017) 84:AEM.01902–01917. 10.1128/AEM.01902-17 PMC5734015 29030448

[21] Jiménez E Ladero V Chico I Maldonado-Barragán A López M Martín V. Antibiotic resistance, virulence determinants and production of biogenic amines among enterococci from ovine, feline, canine, porcine and human milk. BMC Microbiol. (2013) 13:288. 10.1186/1471-2180-13-288 24325647 PMC4029345

[22] Peters J Mac K Wichmann-Schauer H Klein G Ellerbroek L. Species distribution and antibiotic resistance patterns of enterococci isolated from food of animal origin in Germany. Int J Food Microbiol. (2003) 88:311–4. 10.1016/S0168-1605(03)00193-4 14597003

[23] Švec P Franz CMAP The Family Enterococcaceae. (2014). 10.1002/9781118655252.part3

[24] Giraffa G. Functionality of enterococci in dairy products. Int J Food Microbiol. (2003) 88:215–22. 10.1016/S0168-1605(03)00183-1 14596993

[25] Hammad AM Hassan HA Shimamoto T Prevalence, antibiotic resistance and virulence of *Enterococcus*. spp. in Egyptian fresh raw milk cheese. Food Control. (2015) 50:815–20. 10.1016/J.FOODCONT.2014.10.020

[26] Koluman A Akan LS Çakiroglu FP Occurrence and antimicrobial resistance of enterococci in retail foods. Food Control. (2009) 20:281–3. 10.1016/J.FOODCONT.2008.05.007

[27] Agersø Y Lester CH Porsbo LJ Ørsted I Emborg HD Olsen KEP. Vancomycin-resistant *Enterococcus faecalis* isolates from a Danish patient and two healthy human volunteers are possibly related to isolates from imported turkey meat. J. Antimicrob. Chemother. (2008) 62:844–5. 10.1093/jac/dkn271 18586660

[28] Gaglio R Couto N Marques C de Fatima Silva Lopes M Moschetti G Pomba C. Evaluation of antimicrobial resistance and virulence of enterococci from equipment surfaces, raw materials, and traditional cheeses. Int J Food Microbiol. (2016) 236:107–14. 10.1016/J.IJFOODMICRO.2016.07.020 27467501

[29] Jacobsen L Wilcks A Hammer K Huys G Gevers D Andersen SR. Horizontal transfer of tet(M) and erm(B) resistance plasmids from food strains of *Lactobacillus plantarum* to *Enterococcus faecalis* JH2-2 in the gastrointestinal tract of gnotobiotic rats. FEMS Microbiol Ecol. (2007) 59:158–66. 10.1111/j.1574-6941.2006.00212.x 17014680

[30] Ramos S Igrejas G Rodrigues J Capelo-Martinez J-L Poeta P. Genetic characterisation of antibiotic resistance and virulence factors in vanA-containing enterococci from cattle, sheep and pigs subsequent to the discontinuation of the use of avoparcin. Vet J. (2012) 193:301–3. 10.1016/J.TVJL.2011.12.007 22264646

[31] Domann E Hain T Ghai R Billion A Kuenne C Zimmermann K. Comparative genomic analysis for the presence of potential enterococcal virulence factors in the probiotic *Enterococcus faecalis* strain Symbioflor 1. Int J Med Microbiol. (2007) 297:533–9. 10.1016/j.ijmm.2007.02.008 17466591

[32] Franz CMAP Huch M Abriouel H Holzapfel W Gálvez A. Enterococci as probiotics and their implications in food safety. Int J Food Microbiol. (2011) 151:125–40. 10.1016/j.ijfoodmicro.2011.08.014 21962867

[33] Parvez S Malik KA Ah Kang S Kim HY. Probiotics and their fermented food products are beneficial for health. J Appl Microbiol. (2006) 100:1171–85. 10.1111/j.1365-2672.2006.02963.x 16696665

[34] Tuohy KM Probert HM Smejkal CW Gibson GR. Using probiotics and prebiotics to improve gut health. Drug Discov Today. (2003) 8:692–700. 10.1016/S1359-6446(03)02746-6 12927512

[35] Laverde Gomez JA Hendrickx APA Willems RJ Top J Sava I Huebner J. Intra- and interspecies genomic transfer of the Enterococcus faecalis pathogenicity Island. PLoS ONE. (2011) 6:16720. 10.1371/journal.pone.0016720 21559082 PMC3084688

[36] Zühlke D Dörries K Bernhardt J Maaß S Muntel J Liebscher V. Costs of life-Dynamics of the protein inventory of Staphylococcus aureus during anaerobiosis. Sci. Rep. (2016) 6:1–13. 10.1038/srep28172 27344979 PMC4921807

[37] Muntel J Hecker M Becher D. An exclusion list based label-free proteome quantification approach using an LTQ Orbitrap. Rapid Commun Mass Spectrom. (2012) 26:701–9. 10.1002/rcm.6147 22328225

[38] Rappsilber J Mann M Ishihama Y. Protocol for micro-purification, enrichment, pre-fractionation and storage of peptides for proteomics using StageTips. Nat Protoc. (2007) 2:1896–906. 10.1038/nprot.2007.261 17703201

[39] Lassek C Berger A Zühlke D Wittmann C Riedel K. Proteome and carbon flux analysis of *Pseudomonas aeruginosa* clinical isolates from different infection sites. Proteomics. (2016) 16:1381–5. 10.1002/pmic.201500228 26959854

[40] López-Mondéjar R Zühlke D Becher D Riedel K Baldrian P. Cellulose and hemicellulose decomposition by forest soil bacteria proceeds by the action of structurally variable enzymatic systems. Sci Rep. (2016) 6:1–12. 10.1038/srep25279 27125755 PMC4850484

[41] Silva JC Denny R Dorschel CA Gorenstein M Kass IJ Li GZ. Quantitative proteomic analysis by accurate mass retention time pairs. Anal Chem. (2005) 77:2187–200. 10.1021/ac048455k 15801753

[42] Silva JC Gorenstein MV Li G-Z Vissers JPC Geromanos SJ. Absolute quantification of proteins by LCMSE: a virtue of parallel MS acquisition. Mol Cell Proteomics. (2005) 5:144–56. 10.1074/mcp.M500230-MCP200 16219938

[43] Zhang Y Wen Z Washburn MP Florens L Refinements to proteome quantitation based on spectral counting: how to deal with peptides shared by multiple proteins. Anal Chem. (2010) 82:2272–81. 10.1021/ac9023999 20166708

[44] Deutsch EW Csordas A Sun Z Jarnuczak A Perez-Riverol Y Ternent T. The ProteomeXchange consortium in 2017: Supporting the cultural change in proteomics public data deposition. Nucleic Acids Res. (2017) 45:D1100–6. 10.1093/nar/gkw936 27924013 PMC5210636

[45] Schneider T Schmid E de Castro JV Cardinale M Eberl L Grube M. Structure and function of the symbiosis partners of the lung lichen (Lobaria pulmonaria L. Hoffm.) analyzed by metaproteomics. Proteomics. (2011) 11:2752–6. 21604374 10.1002/pmic.201000679

[46] Deutsches Institut für Normung DIN 58940-7 (Standard Draft): Medical Microbiology–Susceptibility Testing of Microbial Pathogens toAntimicrobial Agents–Part 7: Determination of the Minimum Bactericidal Concentration (MBC) by Means of the Microbouillon Dilution Method. Berlin: Beuth (2008).

[47] Carroll RK Rivera FE Cavaco CK Johnson GM Martin D Shaw LN. The lone S41 family C-terminal processing protease in Staphylococcus aureus is localized to the cell wall and contributes to virulence. Microbiol. (2014) 160:1737–48. 10.1099/mic.0.079798-0 24928312 PMC4117222

[48] Thurlow LR Thomas VC Narayanan S Olson S Fleming SD Hancock LE. Gelatinase contributes to the pathogenesis of endocarditis caused by *Enterococcus faecalis*. Infect Immun. (2010) 78:4936–43. 10.1128/IAI.01118-09 20713628 PMC2976315

[49] Semedo T Almeida Santos M Martins P Silva Lopes MF Figueiredeo Marques JJ Teneiro R Comparative study using type strains and clinical and food isolated to examine hemolytic activity and ocurrente of the cyl operon in enterococci. J Clin Microbiol. (2003) 41:2569–76. 10.1128/JCM.41.6.2569-2576.2003 12791882 PMC156526

[50] Dupont H Montravers P Mohler J Carbon C. Disparate findings on the role of virulence factors of *Enterococcus faecalis* in mouse and rat models of peritonitis. Infect Immun. (1998) 66:2570–75. 9596718 10.1128/iai.66.6.2570-2575.1998 PMC108240

[51] Rich JJ Kinscherf TG Kitten T Willis DK. Genetic evidence that the gacA gene encodes the cognate response regulator for the lemA sensor in *Pseudomonas syringae*. J Bacteriol. (1994) 176:7468–75. 10.1128/jb.176.24.7468-7475.1994 8002569 PMC197202

[52] Williamson R Le Bouguenec C Gutmann L Horaud T. One or two low affinity penicillin-binding proteins may be responsible for the range of susceptibility of *Enterococcus faecium* to benzylpenicillin. J Gen Microbiol. (1985) 131:1933–40. 10.1099/00221287-131-8-1933 3850924

[53] Lynch C Courvalin P Nikaido H. Active efflux of antimicrobial agents in wild-type strains of Enterococci. Antimicrob. Agents Chemother. (1997) 41:869–71. 9087510 10.1128/aac.41.4.869 PMC163815

[54] Brown DFJ Reynolds PE Intrinsic resistance to /3-lactam antibiotics in *Staphylococcus aureus*. (1980) 122:275–8. 10.1016/0014-5793(80)80455-8 7202719

[55] Hollenbeck BL Rice LB. Intrinsic and acquired resistance mechanisms in enterococcus. Virulence. (2012) 3:421–33. 10.4161/viru.21282 23076243 PMC3485979

[56] Galloway-Peña JR Nallapareddy SR Arias CA Eliopoulos GM Murray BE. Analysis of clonality and antibiotic resistance among early clinical isolates of *Enterococcus faecium* in the United States. J Infect Dis. (2009) 200:1566–73. 10.1086/644790 19821720 PMC2784011

[57] Montealegre MC Roh H Rae M Davlieva MG Singh KV Shamoo Y. Differential Penicillin-Binding Protein 5 (PBP5) levels in the *Enterococcus faecium* clades with different levels of ampicillin resistance. Antimicrob Chemother. Chemother. (2017) 61:1–10. 10.1128/AAC.02034-16 27821450 PMC5192164

[58] Ono S Muratani T Matsumoto T. Mechanisms of resistance to imipenem and ampicillin in *Enterococcus faecalis*. Antimicrob Agents Chemother. (2005) 49:2954–8. 10.1128/AAC.49.7.2954-2958.2005 15980374 PMC1168717

[59] Wright GD. Bacterial resistance to antibiotics: enzymatic degradation and modification. Adv Drug Deliv Rev. (2005) 57:1451–70. 10.1016/j.addr.2005.04.002 15950313

[60] Fontana R Canepari P Lleò MM Satta G. Mechanisms of resistance of enterococci to beta-lactam antibiotics. Eur J Clin Microbiol Infect Dis. (1990) 9:103–5. 2180705 10.1007/BF01963633

[61] Temmerman R Pot B Huys G Swings J. Identification and antibiotic susceptibility of bacterial isolates from probiotic products. Int J Food Microbiol. (2003) 81:1–10. 10.1016/S0168-1605(02)00162-9 12423913

[62] Wacher-Rodarte M del C Trejo-Muñúzuri TP Montiel-Aguirre JF Drago-Serrano ME Gutiérrez-Lucas RL Antibiotic resistance and multidrug-resistant efflux pumps expression in lactic acid bacteria isolated from pozol, a nonalcoholic Mayan maize fermented beverage. Food Sci Nutr. (2016) 4:423–30. 10.1002/fsn3.304 27247772 PMC4867762

[63] Cookson BD. The emergence of mupirocin resistance: a challenge to infection control and antibiotic prescribing practice. J Antimicrob Chemother. (1998) 41:11–8. 9511032 10.1093/jac/41.1.11

[64] Ruiz-Garbajosa P Bonten MJM Robinson DA Top J Nallapareddy SR Torres C. Multilocus sequence typing scheme for *Enterococcus faecalis* reveals hospital-adapted genetic complexes in a background of high rates of recombination. J Clin Microbiol. (2006) 44:2220–8. 10.1128/JCM.02596-05 16757624 PMC1489431

[65] Dunny GM Leonard BAB. Cell-cell communication in gram-positive bacteria. Annu Rev Microbiol. (1997) 51:527–64. 10.1146/annurev.micro.51.1.527 9343359

[66] Fisher K Phillips C. The ecology, epidemiology and virulence of Enterococcus. Microbiology. (2009) 155:1749–57. 10.1099/mic.0.026385-0 19383684

[67] An FY Sulavik MC Clewell DB Identification and characterization of a determinant (eep) on the *Enterococcus faecalis* chromosome that is involved in production of the peptide sex pheromone. J Bacteriol. (1999) 81:5915–21. 10.1128/jb.181.19.5915-5921.1999 PMC103617 10498702

[68] Posch S Aponte-Santamaría C Schwarzl R Karner A Radtke M Gräter F. Mutual A domain interactions in the force sensing protein von Willebrand factor. J Struct Biol. (2017) 197:57–64. 10.1016/J.JSB.2016.04.012 27113902

[69] Konto-Ghiorghi Y Mairey E Mallet A Duménil G Caliot E Trieu-Cuot P. Dual role for pilus in adherence to epithelial cells and biofilm formation in *Streptococcus agalactiae*. PLoS Pathog. (2009) 5:e1000422. 10.1371/journal.ppat.1000422 19424490 PMC2674936

[70] Reffuveille F Leneveu C Chevalier S Auffray Y Rincé A. Lipoproteins of Enterococcus faecalis: Bioinformatic identification, expression analysis and relation to virulence. Microbiology. (2011) 157:3001–13. 10.1099/mic.0.053314-0 21903750

[71] Jones BV Begley M Hill C Gahan CGM Marchesi JR. Functional and comparative metagenomic analysis of bile salt hydrolase activity in the human gut microbiome. Proc Natl Acad Sci USA. (2008) 105:13580–5. 10.1073/pnas.0804437105 18757757 PMC2533232

[72] Begley M Hill C Gahan CGM. Bile salt hydrolase activity in Probiotics. Appl Environ Microbiol. (2006) 72:1729–38. 10.1128/AEM.72.3.1729 16517616 PMC1393245

[73] Le Breton Y Mazé A Hartke A Lemarinier S Auffray Y Rincé A Isolation and characterization of bile salts-sensitive mutants of *Enterococcus faecalis*. Curr Microbiol. (2002) 45:434–9. 10.1007/s00284-002-3714-9 12402085

[74] Genovese F Coïsson JD Majumder A Pessione A Svensson B Jacobsen S An exoproteome approach to monitor safety of a cheese-isolated *Lactococcus lactis*. Food Res Int. (2013) 54:1072–9. 10.1016/J.FOODRES.2012.12.017

[75] Sánchez B Urdaci MC Margolles A. Extracellular proteins secreted by probiotic bacteria as mediators of effects that promote mucosa-bacteria interactions. Microbiology. (2010) 156:3232–42. 10.1099/mic.0.044057-0 20864471

